# Vitamin A in Reproduction and Development

**DOI:** 10.3390/nu3040385

**Published:** 2011-03-29

**Authors:** Margaret Clagett-Dame, Danielle Knutson

**Affiliations:** 1 Department of Biochemistry, University of Wisconsin-Madison, 433 Babcock Drive, Madison, WI 53706, USA; Email: dcknutson@wisc.edu; 2 School of Pharmacy, Pharmaceutical Sciences Division, University of Wisconsin-Madison, 777 Highland Ave., Madison, WI 53705, USA

**Keywords:** retinoic acid, vitamin A deficiency, embryonic

## Abstract

The requirement for vitamin A in reproduction was first recognized in the early 1900’s, and its importance in the eyes of developing embryos was realized shortly after. A greater understanding of the large number of developmental processes that require vitamin A emerged first from nutritional deficiency studies in rat embryos, and later from genetic studies in mice. It is now generally believed that all-*trans* retinoic acid (RA) is the form of vitamin A that supports both male and female reproduction as well as embryonic development. This conclusion is based on the ability to reverse most reproductive and developmental blocks found in vitamin A deficiency induced either by nutritional or genetic means with RA, and the ability to recapitulate the majority of embryonic defects in retinoic acid receptor compound null mutants. The activity of the catabolic CYP26 enzymes in determining what tissues have access to RA has emerged as a key regulatory mechanism, and helps to explain why exogenous RA can rescue many vitamin A deficiency defects. In severely vitamin A-deficient (VAD) female rats, reproduction fails prior to implantation, whereas in VAD pregnant rats given small amounts of carotene or supported on limiting quantities of RA early in organogenesis, embryos form but show a collection of defects called the vitamin A deficiency syndrome or late vitamin A deficiency. Vitamin A is also essential for the maintenance of the male genital tract and spermatogenesis. Recent studies show that vitamin A participates in a signaling mechanism to initiate meiosis in the female gonad during embryogenesis, and in the male gonad postnatally. Both nutritional and genetic approaches are being used to elucidate the vitamin A-dependent pathways upon which these processes depend.

## 1. Background

**Figure 1:**
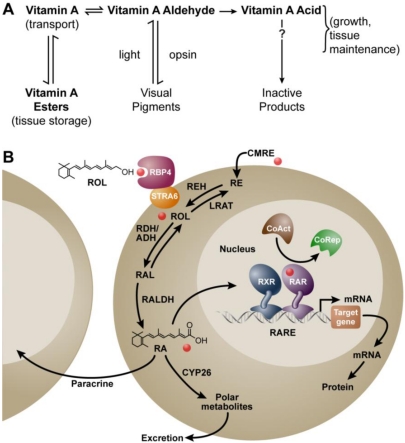
Metabolism of vitamin A (retinol) to all-*trans* retinoic acid (RA), and the mechanism of RA action. (**A**) Metabolic scheme proposed by Dowling and Wald in 1960 [11]; (**B**) Mechanism *ca.* 2011. Vitamin A (retinol, ROL) circulates bound to the plasma retinol-binding protein (RBP4) and transthyretin (not shown). RBP4 binds to the membrane receptor STRA6 to facilitate the cellular uptake of retinol in some cells. Vitamin A circulating as part of a chylomicron remnant (CMRE) can also serve as a source of vitamin A for the cell. Note that cellular retinol and RA binding proteins have been omitted for simplicity. Retinol is either esterified by lecithin:retinol acyltransferase (LRAT) and stored, or is oxidized reversibly to retinaldehyde (RAL) by retinol dehydrogenases (RDH/ADH), and further oxidized in irreversible fashion to RA by retinaldehyde dehydrogenase (RALDH 1, 2, or 3). In the nucleus, the RAR/RXR complex is bound to a specific sequence of DNA called the retinoic acid response element (RARE). Binding of RA to the RAR leads to release of the corepressor complex (CoRep) and association with coactivator proteins (CoAct), followed by altered transcription of downstream target genes and ultimately changes in cellular function. RA also undergoes further oxidation by the cytochrome P450 (CYP) 26 family to more polar metabolites. The lipophilic molecule, RA, can act within the same cell in which it is synthesized (autocrine) or can diffuse through the cell membrane to act in nearby cells (paracrine). Abbreviations: ADH, alcohol dehydrogenase; RDH, retinol dehydrogenase; REH, retinyl ester hydrolase; RE, retinyl ester.

It has been nearly 100 years since the essential micronutrient, vitamin A, was first described. In 1913 McCollum and Davis reported that the addition of an ether extract from egg yolk or butter, but not lard or olive oil, could reinstate growth in rats maintained for several months on a purified ration of casein, carbohydrates and salt mixtures [1]. We now know that this essential accessory article in foodstuffs is vitamin A. Using dietary manipulation to induce deficiency in rats, the importance of vitamin A in both male and female reproduction was soon discovered [2,3]. For a time, there was confusion over how both yellow-colored foods and those that were colorless, for example extract from pork liver or cod liver oil, could both yield vitamin A activity [4]. This problem was solved when Moore fed carotenoids to vitamin A-deficient (VAD) rats in amounts that enabled the animals to resume normal growth, and showed that only the “colorless” form of vitamin A was found in the livers collected from these animals [5]. Thus, it was realized that carotenoids (at least a subset) could be converted to vitamin A, a fact that was fully appreciated when Karrer *et al.* published the chemical structures of both carotene and vitamin A [6,7]. In 1946, Arens and van Dorp synthesized vitamin A acid (retinoic acid), and reported it was as potent as vitamin A in supporting the growth of VAD rats but could not be converted back to vitamin A [8,9,10]. The metabolic scheme in which vitamin A (retinol) generates the vitamin A aldehyde (retinaldehyde) to support synthesis of the visual pigments, and its further irreversible oxidation to the vitamin A acid (all-*trans* retinoic acid, RA) that supports growth and tissue maintenance was first reported in the landmark paper by Dowling and Wald and this metabolic scheme stands essentially unchanged today (Figure 1A) [11]. 

All-*trans* retinol (retinol, vitamin A) is obtained in the diet from plant sources (carotenoids with vitamin A activity) or as retinyl esters from animal sources. Retinol has two major fates: (1) esterification and tissue storage, and (2) oxidative metabolism to all-*trans* retinaldehyde and further oxidation to RA. The enzyme lecithin:retinol acyltransferase (LRAT) is responsible for esterifying the majority of retinol into retinyl esters [12]. The first and rate-limiting step in the production of RA from retinol results from the action of cytosolic alcohol dehydrogenases (ADH) and microsomal retinol dehydrogenases (RDH) yielding all-*trans* retinaldehyde [13]. The irreversible oxidation of all-*trans* retinaldehyde to all-*trans* retinoic acid is catalyzed by several aldehyde dehydrogenases (RALDH), of the ALDH1A class (ALDH 1A1, 1A2, and 1A3 also known as RALDH 1, 2 and 3) [14,15]. RALDH-independent generation of RA from retinol by CYP1B1 has also been reported [16]. The metabolism of RA at the C4 and C18 positions to oxidative metabolites including 4-hydroxy-RA, 18-hydroxy-RA, and 4-oxo-RA occurs by the action of cytochrome P450 enzymes of the CYP26 family (A1, B1 and C1) [17,18,19,20,21,22,23]. Vitamin A and metabolites are lipophilic compounds that are generally found in association with serum and cellular binding proteins [24]. Retinol-binding protein (RBP or RBP4) carries the majority of retinol in the circulation [25], and a membrane receptor, STRA6, binds to RBP to enable efficient retinol uptake by a number of cells [26]. In addition, cellular proteins that bind to retinol (CRBP I, II, and III), and RA (CRABP I and II) have been studied in null mutant mice; some have been found to be dispensable, while roles for others have been revealed when animals are fed a vitamin A-restricted diet [12,27,28].

RA is a ligand for the nuclear retinoic acid receptor (RAR) proteins. There are three major subtypes of RAR protein (α, β and γ) with additional isoforms resulting from alternate promoter usage and splicing [29]. The nuclear RARs act as ligand-activated transcription factors to regulate gene transcription in a cell-type and tissue-specific manner [30]. The all-*trans* isomer of RA is the highest affinity endogenous ligand for the RAR [31]. Members of a second protein family, the retinoid (rexinoid)-X receptors (RXR) heterodimerize with RAR to confer high-affinity binding to DNA. The DNA to which the RAR/RXR heterodimer binds is called a retinoic acid response element (RARE). The consensus RARE is composed of two direct repeats of PuG(G/T)TCA separated most often by 5 bases [32]. However, more complex elements that do not adhere to this rule have been described [33]. RAREs serve as enhancer elements and when occupied by the RA/RAR/RXR complex, facilitate chromatin opening and changes in RA target gene transcriptional activity [34,35]. A large number of genes are altered when cells or tissues are exposed to RA, however, only a small subset are primary (direct) targets via RARE-mediated transcription, with the remainder representing downstream targets [36,37]. A schematic summarizing the metabolism of vitamin A and the cellular mechanism of RA action is shown in Figure 1B. Note in this review retinoid is a term that refers to compounds structurally-related to retinol, and in this review, is used to refer to vitamin A and its metabolites.

## 2. Vitamin A and Reproduction

### 2.1. Male Reproduction

**Figure 2:**
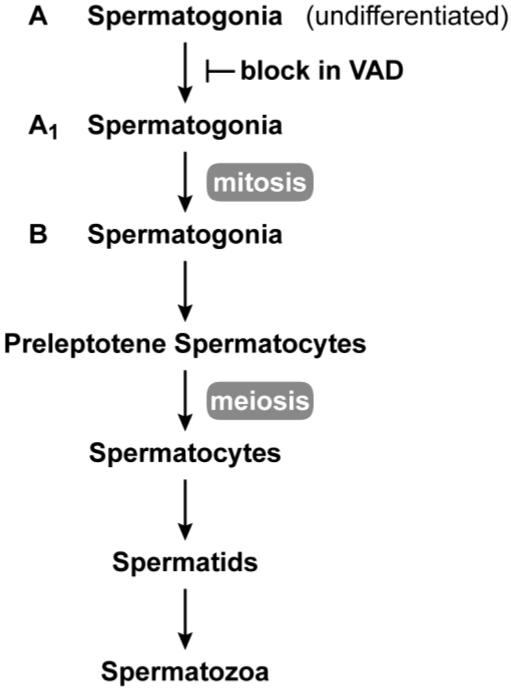
Spermatogenesis in the adult. Spermatogenesis takes place in the seminiferous epithelium of testis tubules from puberty through adulthood. Undifferentiated (A-type) spermatogonia at the base of the seminiferous epithelium divide mitotically until they enter the differentiation pathway to become A1 spermatogonia. A1 spermatogonia undergo division to A1-A4 and finally B spermatogonia. B spermatogonia divide to produce preleptotene (primary) spermatocytes that migrate away from the base of the seminiferous tubule to undergo meiosis. The first meiotic division produces secondary spermatocytes, and after the second meiotic division, spermatids (haploid cells) begin the differentiation process (spermiogenesis) to spermatozoa. In vitamin A deficiency, the transition from A to A1 spermatogonia is blocked [42,43,44].

Vitamin A is required for male reproduction. Early work in the laboratories of Wolbach and Howe as well as Mason showed that in vitamin A deficiency, the epithelia of the epididymis, prostate, and seminal vesicle is replaced with stratified squamous keratinizing epithelium, and spermatogenesis ceases [2,38]. Later work showed that in the VAD rat testes, undifferentiated spermatogonia, Sertoli cells and a small number of preleptotene spermatocytes remain [39,40,41], whereas in the mouse, spermatogenesis is arrested at the spermatogonia stage [42]. Upon addition of vitamin A, spermatogenesis can be reinstituted by stimulating A to A1 spermatogonial differentiation in a synchronized manner [42,43,44]. The block in adult spermatogenesis resulting from vitamin A deficiency is shown in Figure 2. Recent work supports the conclusion that the vitamin A metabolite, RA, is needed both for adult male spermatogonial differentiation (transition to A1) and the entrance into meiosis [45,46,47]. 

In 1991, Van Pelt and de Rooij determined that a large dose of RA (5 mg) given by injection twice a week, when combined with a RA-containing diet, supported the development of spermatocytes, and their subsequent development into spermatids in VAD rats supporting that RA is the active form of vitamin A in male reproduction [48]. A CYP26-mediated catabolic barrier comprised of peritubular myoid cells surrounds the seminiferous tubule, and may prevent RA in the general circulation from reaching cells in the interior of the tubule, thus explaining why such high doses of exogenous RA were required [49,50]. Within the normal tubule, the Sertoli cell is believed to generate RA by the action of *Raldh1* [49,51,52], and possibly *Raldh2*[50]. *Raldh2* is also found in late pachytene and diplotene spermatocytes, and early stage spermatids. 

RARγ null males are sterile and exhibit squamous metaplasia of the seminal vesicles and prostate glands [53]. RARα null mutants are sterile and show a reduction in spermatozoa, indicating that nuclear RAR is needed for spermiation [54]. RARα is expressed primarily in the Sertoli cell, *Rarβ* in spermatids and RARγ in A spermatogonia [49]. Expression of RARα is needed for differentiation of the spermatogonia during prepuberty [55]. However, RAR signaling in the Sertoli cell cannot account for the VAD-induced arrest in spermatogonia differentiation, as deletion of all RARs in the Sertoli cell does not cause arrest of spermatogonia differentiation in the adult mouse. The cell types in which RA and its receptors act to support spermatogenesis continues to be a subject of active investigation [45,49,55,56,57]. 

### 2.2. Female Reproduction

In the female, the effect of vitamin A deficiency on reproductive outcome is dependent upon the time when deficiency is imposed, as well as its severity [58]. When severe vitamin A deficiency is imposed prior to mating, cornified cells are continuously present in vaginal smears [59,60] and reproduction fails prior to implantation [3]. VAD female rats continue to ovulate and form corpora lutea irregularly or at normal intervals, however, degenerated eggs are found in the last portion of the tube, and there is no evidence that blastogenesis has occurred.

Warkany and Schraffenberger showed that when limited amounts of provitamin A carotenoid are provided to VAD female rats prior to mating, a less severe maternal vitamin A deficiency is produced which enables fertilization and implantation to occur, but embryonic death at midgestation often results [61]. If provided in adequate amounts, retinol will support reproduction and embryonic development in full [62]. RA in amounts ranging from 2 to 12 mcg/g diet or 40 to 230 mcg/rat/day given to a VAD female rat is sufficient to maintain normal fertilization, implantation and early embryogenesis. However, pregnant animals maintained on this level of RA will invariably resorb all the fetuses. Higher amounts of RA (250 mcg/g diet or approximately 4.5 mg/rat/day) or retinol is needed by embryonic day 8.5 (E8.5) (late gastrula/early neurula) to support normal embryonic development and overcome midgestational resorption [63,64,65,66,67].

Maternal vitamin A also plays a role in placental development and/or maintenance, as the chorioallantoic placenta undergoes widespread necrosis by E15.5 in VAD rats supported on insufficient amounts of RA [68]. Microscopic analysis of the placenta from E14 to E17 reveals changes in the central region of the junctional zone and the labyrinthine zone of VAD rats supported on insufficient vitamin A acid, whereas addition of retinyl acetate to the diet prevents these changes [69]. Microscopic findings suggest the differentiation of the parenchymal cells of the junctional zone to glycogen cells, and of the chorionic trophoblast cells to the inner trophoblastic lamina of the trichorial placenta is affected. In summary, the relative vitamin A status of the female, both at the time of conception and throughout pregnancy, is a critical determinant in reproductive outcome, and deficiency can lead to either a complete failure of reproduction prior to implantation or fetal resorption or malformation.

### 2.3. Germ Cell Development

**Figure 3:**
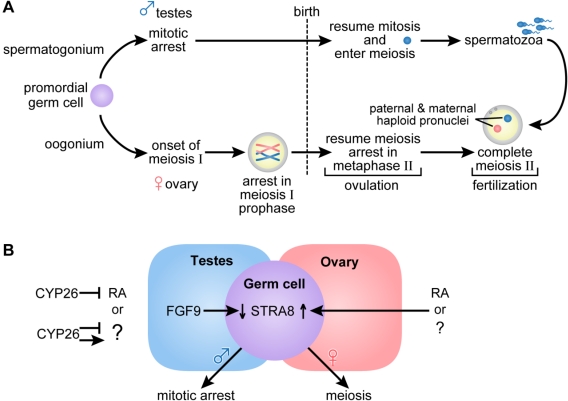
(**A**) Germ cell development and gametogenesis. Primordial germ cells colonize the gonad in both male and female embryos. The first morphological marker of sex-specific germ cell development is seen in the female embryo when the oogonia enter meiosis. Primary oocytes proceed through the leptotene, zygotene and pachytene stages of meiotic prophase before birth, when they arrest in diplotene of meiosis I. At ovulation, meiosis I is completed, and the secondary oocytes enter meiosis II and arrest again in metaphase. Meiosis II is completed after fertilization. In the male embryo, germ cells are committed to the spermatogenic program but arrest in G0/G1, and do not complete mitosis and enter meiosis until after they are born. Primary spermatocytes entering meiosis I are seen during the first week of life; secondary spermatocytes complete meiosis II forming spermatids and functional gametes called spermatozoa or sperm. In the male, waves of meiosis continue throughout life. (**B**) In the female embryo access to RA or alternatively, another factor indicated as (?), promotes entry into meiosis whereas embryonic male germ cells are maintained in a pluripotent state. Either RA or another factor acts in the embryonic female germ cell to increase *Stra8*, essential for entry into meiosis. In the ovary, *Fgf9* levels are low. In the male embryo, entry into meiosis is prevented by the action of CYP26B1; high levels of *Fgf9* in the testes antagonize *Stra8* expression and maintain germ cells in a pluripotent state. (Adapted from [77,78]).

The generation of sperm and oocytes requires germ cells to undergo meiosis, the process in which diploid cells give rise to haploid cells. In the female, germ cells enter meiosis I during embryogenesis, whereas in the male, this process occurs postnatally (Figure 3A). It has been proposed that access of primordial germ cells (gonocytes) to RA plays an important role in determining when they will enter meiosis, with female germ cells entering into meiosis after exposure to embryonic RA, whereas, in the male embryo, this pathway is blocked by the action of CYP26B1, but is enabled after birth [70,71,72,73]. However, a report appearing when this manuscript was under review raises questions concerning a role for RA in this model [74].

The culture of embryonic rat ovaries with RA promotes meiotic entry [75], whereas murine embryonic ovaries (E11.5) cultured in the presence of the RAR panantagonist BMS-204493 for 2 days do not express the RA-responsive gene *Stra8* [70], a gene that is required for meiotic initiation in female gonocytes [76]. However, a recent report shows that female RALDH2 and RALDH2/3 knockout gonads express *Stra8* and undergo meiosis in the absence of detectable RA activity as assessed using a *β2-RARE-lacZ* (*RARE-lacZ*) reporter [74]. If the reporter accurately detects all RA activity, this new work indicates that meiotic initiation can occur independently of RA signaling. 

There is clear *in vivo* evidence that vitamin A is required for the normal onset of meiotic prophase in ovarian germ cells [79]. While fetal ovarian germ cell number is unaffected by vitamin A deficiency, the germ cells in embryos with the most severe vitamin A deficiency fail to enter meiosis as evidenced by a lack of immunostaining for SYCP3 (a gene that encodes a component of the synaptonemal complex), and the critical RA-responsive gene, *Stra8* is nearly undetectable. When RA is included in the maternal diet at a slightly higher level, but one insufficient to support most other vitamin A-dependent embryonic processes, a small number of cells undergo meiosis (30%) compared to 75% of cells in the vitamin A-sufficient group. Thus, it is possible that only a very low level of RA is needed to initiate meiosis, or alternatively, vitamin A may support meiotic entry by an alternative mechanism.

The mesonephros has been proposed as the source of RA that initiates the entry of primordial gonocytes into meiosis [71]. *Raldh2* is largely responsible for RA synthesis in the mesonephros, with some contribution from *Raldh3* in the region of the mesonephric duct [71,80]. Expression of *Raldh1* mRNA is reported in the adjacent male gonad from E11.5 to 13.5, and to a lesser extent in the female gonad by E13.5 [81]. The gene encoding the RA-metabolizing cytochrome P450 enzyme, *Cyp26b1*, is expressed early in the genital ridge, but is decreased after E12.5 in the female mouse embryo, thus enabling access of the gonocyte to RA [71]. In contrast, the expression of *Cyp26b1* persists in the male embryonic gonad, and has been proposed to prevent RA from stimulating the gonocytes to undergo meiosis [70,71]. Embryonic male germ cells undergo G_0_/G_1_ mitotic cell cycle arrest and do not enter meiosis until a week after birth. In CYP26B1 null mutant male embryos, RA levels in the testes are increased and germ cells prematurely enter meiosis at embryonic day 13.5, and this is followed by apoptosis [73]. CYP26B1 is also required later in embryogenesis to maintain cells in an undifferentiated state [82]. However, a new report shows that inhibition of CYP26 activity with ketoconazole results in a similar increase in *Stra8* mRNA in cultured wild-type and RALDH2 null mutant testis/mesonephros complexes, whereas no expression is seen in testes cultured in isolation [74]. This suggests that some factor other than RA from the mesonephros may be affected by CYP26, and thus control entry into meiosis. Thus, either a very low level of RA that is undetectable by the *RARE-lacZ* reporter and that is generated by an enzyme other than RALDH2 is responsible for meiotic entry, or alternatively, CYP26B1 functions by degrading an unknown inducer of *Stra8* or participates in the synthesis of a factor that inhibits *Stra8* expression. 

Recently, Li *et al.* showed that vitamin A is required in the male neonate for germ cells to enter the first round of spermatogenesis [83]. Vitamin A deficiency was produced in prepubertal life by maintaining LRAT^−/−^ female mice on a diet devoid of all vitamin A during pregnancy and lactation. It was possible to generate deficiency at this time because LRAT^−/−^ mice cannot store retinol in the form of retinyl esters in most tissues, and thus the mothers and pups can be more rapidly depleted of their vitamin A stores than wild-type mice. In the wild-type mouse, *Stra8* begins to increase in the testis by postnatal day 6 [84] and spermatogenesis begins shortly thereafter [85]. However, in the VAD LRAT^−/−^ gonad the expected increase in *Stra8* did not occur. Germ cells, although present in normal numbers, did not undergo meiosis as was observed in the vitamin A-sufficient controls and instead remained undifferentiated [83]. The addition of retinol to VAD LRAT^−/−^ pups at postnatal day 5 prevented meiotic failure. Thus, it appears that an environment depleted of vitamin A maintains germ cells in an undifferentiated state, and vitamin A sufficiency enables entry into meiosis I in both the developing female and male gonad. 

## 3. Vitamin A and Embryonic Development

### 3.1. Embryonic Vitamin A Deficiency Studies

Very early evidence that vitamin A is required for embryonic development came from reports of abnormalities in pigs born to gilts on a VAD diet [86,87]. A range of reproductive outcomes were reported in gilts fed a vitamin A-free ration from 160 days before breeding and for 30 days thereafter. Some gilts on the deficient diet failed to show the symptoms of estrous, whereas others became pregnant, but resorbed all the fetuses. The remaining offspring from litters that survived to term showed a lack of eye development or no eyes. Cleft palate, accessory ears and arrested ascension of the kidneys were also observed, but at a lower frequency. In the 1940’s, Warkany and colleagues published their landmark studies describing a syndrome of defects in rat embryos from VAD mothers given limited amounts of carotene [61,88,89,90,91,92,93,94,95]. Approximately 70% of the VAD female rats supplemented in this fashion conceived, however, the majority of pregnancies did not continue beyond midgestation [61]. Ocular defects were most frequently observed in offspring from the few pregnancies that progressed to term. In order to understand how maternal vitamin A deficiency affected development, fetuses ranging from embryonic days 12.5 to 20.5 (near term) were taken by cesarean section whenever evidence of maternal bleeding was observed, and histology was performed. Of the fetuses recovered in this fashion, defects in eye development were most frequent (49% with one or more abnormality) and included coloboma, retinal eversion, penetration of the retina by mesodermal tissue, low insertion of the optic stalk and the cup, and defects in the iris. Defects at lower penetrance were noted in other systems including the genitourinary tract (42%), kidney (38%), diaphragm (31%), lung (4%), aortic arch (9%) and heart (4%) [95]. The low penetrance of many of these defects likely resulted from variation both in the timing and severity of maternal vitamin A deficiency. 

Taking advantage of the fact that RA will support early fetal development in VAD rats [65], See *et al.* was able to generate the previously described vitamin A deficiency syndrome in 100% of fetuses (with the exception of cardiovascular defects) from mothers supported on a sufficient amount of RA up to E10.5, and fed a suboptimal amount of RA thereafter (late VAD rat model, Table 1) [67]. Rapid initiation of VAD is possible using this approach because RA, unlike retinol, is not stored and has a very short biological half-life. The low penetrance of cardiovascular defects was due to institution of deficiency after aortic arch and septal development had largely been completed. The addition of retinol after E10.5 prevented all fetal anomalies from appearing, and the addition of a higher level of RA led to either a complete or partial rescue of fetal anomalies, supporting the idea that RA is the functional form of vitamin A in embryonic development. Using nutritional models in which RA deficiency is imposed either early, or after the 12-15 somite stage, a number of defects have been added to the original fetal vitamin A deficiency syndrome. Nervous system, cardiovascular, and axial patterning defects result from early deficiency [63,64,65,66,96], whereas a less well-developed nasal region, salivary gland hypoplasia, agenesis of the Harderian glands, hypoplasia of the intestinal villi and a number of skeletal abnormalities arise if RA is limiting at later times [67].

**Table 1:** Summary of abnormalities in VAD rat embryos given insufficient RA (late VAD) * [67], insufficient carotene (VAD syndrome) [95], or observed in RAR null mutants [97,98,99].

	Late VAD	VAD syndrome	Observed in RAR null mutants
Ocular	100%	49%	Yes
*Types*			
Eversion of retina	100%	27%	Yes
Fibrous retrolenticular membrane	100%	49%	Yes
Coloboma	100%	18%	Yes
Heart-interventricular septal defect	17%	4%	Yes
Lung-hypoplasia	100%	4%	Yes
Diaphragmatic hernia	100%	31%	Yes
Intestinal villi hypoplastic	83%	Not reported	Not reported
Kidney	100%	38%	Yes
*Types*			
Kidneys too close or fused	100%	20%	Not reported
Ectopia	100%	4%	Yes
Ureter-ectopic termination	100%	36%	Yes
Undescended testes	100%	54%	Not reported
Skeletal defects	100%	Not reported	Yes
Glandular defects	100%	Not reported	Yes
Nasal region less developed	100%	Not reported	Yes

* Dietary RA restricted after E10.5 (12-15 somite stage) of development.

### 3.2. Role of the Retinoic Acid Receptors

The *Rars* are widely expressed in development [100]. A series of genetic experiments revealed the importance of the RAR in mediating the actions of RA in developing embryos [99,101,102]. These key experiments showed that compound RAR mutants die either *in utero* or shortly after birth, and recapitulate most of the defects described as part of the vitamin A deficiency syndrome [61,88,89,90,91,92,93,94,95,103] and the late VAD rat embryo model [67,104]. These genetic studies provide clear evidence that the vitamin A metabolite, RA, acts through a RAR signaling mechanism in support of normal embryonic development. 

RAR/RXR heterodimers form the functional unit that transduces the RA signal, with one genomic copy of the *Rxrα* sufficient to support RXR function in embryogenesis [105]. Ocular defects are present in some RXRα mutants lacking only the AF-2 domain (activation function) in the ligand-binding domain [99,106,107]. However, work from the Duester group indicates that ligand binding to the RAR is sufficient to transduce the signal, as a RAR- but not a RXR- selective ligand is able to rescue the developmental defects in RALDH2 null mutant embryos [108]. Thus, the need for a RXR ligand in RAR-driven events remains to be established.

### 3.3. Transport of Retinoid from Maternal to Fetal Compartment

There are at least two ways in which retinoid is transferred from the maternal blood to the fetus. The major form of transport is the binding of retinol to a specific binding protein (RBP or RBP4) [109,110]. Retinyl esters can also be transported in the form of chylomicron remnants or as a part of very low-density and low-density lipoproteins. During early placentation, RBP is localized in the endoderm of the yolk sac visceral wall surrounding the embryo, and after definitive placentation, in the yolk sac membranes as well as in the uterus (decidua basalis) [111,112]. RBP does not cross the placenta into the fetal circulation [113]. Maternal retinol must be transferred to fetal RBP [114], or alternatively, retinol may be esterified in the placenta, and delivered to tissues in lipoproteins [115,116]. Although RBP null mutant embryos from null mutant mothers develop normally on a vitamin A-sufficient diet, embryogenesis is perturbed when maternal vitamin A intake is restricted [110]. Thus, when RBP is not present, circulating retinyl ester appears to represent the main pathway for the provision of retinoid from mother to embryo, provided maternal vitamin A intake is adequate [113].

### 3.4. Embryonic RA Synthesis and Catabolism

It is clear that RA is essential for embryonic development. However, too much RA at critical stages can result in embryo lethality or malformation [117,118,119,120]. Thus, regulation of the amount of RA that is available to the embryo at specific times and to a given tissue site is of critical importance. Both the distribution and function of enzymes involved in RA synthesis and degradation have been studied intensively in developing embryos. Interestingly, both the levels and domains of *Cyp26* gene expression in the developing embryo are affected by retinoid status, whereas the *Raldhs* are unaffected [121]. 

Of the retinol dehydrogenase enzymes, RDH10 plays a key role, as loss results in embryonic lethality by approximately E13.0 [122]. These embryos (trex mutants) show a spectrum of defects consistent with vitamin A deficiency, although they do not die as early in gestation as severely RA-depleted embryos [65,123], indicating that one or more additional enzymes must also be active in generating retinaldehyde for RA synthesis. Supplementation with RA rescues the abnormalities, supporting a role for RDH10 in RA biosynthesis. A recent detailed examination of *Rdh10* in the avian embryo shows that the expression domain is often smaller than that of the corresponding *Raldh*, leading the authors to suggest that retinaldehyde may be transferred between cells [124]. Recent work in *Xenopus* shows that RA down regulates *XRDH10* transcripts, and this may serve as an additional mechanism to regulate endogenous retinoid levels [125]. *Adh 1*, *3* and *4* enzyme family members are also expressed in developing embryos [126,127], however, mutation does not result in embryo lethality [128,129]. It should be noted, that ADH3 and ADH4 null mutant mice do undergo early postnatal lethality if maintained for two generations on a VAD diet [129]. Thus, to date, RDH10 is the only retinol dehydrogenase shown to be indispensable for embryonic development under normal dietary conditions.

Numerous retinaldehyde dehydrogenase single and compound deletion mutants have been generated and studied [123,130,131,132,133,134,135]. Deletion of RALDH2 is lethal early in development, whereas RALDH3 null mutants die at birth [131], and RALDH1 mutants are viable [133]. Thus, RALDH2 is the earliest expressed family member to produce RA in the embryo, and is believed responsible for all RA signaling activity from E7.5-E8.5 in the mouse [136]. Shortly thereafter, RALDH1 and RALDH3 contribute to RA synthesis in the eye and olfactory pit. *Cyp1B1* can also generate RA and is expressed at known sites of RALDH-independent retinoid signaling [16], however, null mutant mice for this gene are viable [137]. Recent work in zebrafish indicates that the availability of retinaldehyde for RA synthesis may represent another point of control, with a short-chain dehydrogenase/reductase family member, DHRS3a, which catalyzes reduction of retinaldehyde to retinol serving as a RA-induced feedback inhibitor of RA biosynthesis [138].

The importance of CYP26 enzymes in restricting RA distribution and availability is illustrated by studies in which one or more is genetically ablated. Mutation of *Cyp26A1* is embryonic lethal and mutants die during mid- to late gestation with symptoms mirroring those observed in RA toxicity including caudal defects and occasionally exencephaly [139,140,141]. CYP26B1 null mutants die immediately after birth and exhibit limb, male germ cell, and craniofacial abnormalities [73,142,143]; whereas loss of CYP26C1 alone does not produce an overt phenotype [144]. Deletion of both mouse CYP26 A1 and C1 is lethal at E9.5 to E10.5 [144], and deletion of all three CYP26 family members in the mouse is also lethal early in embryonic life, and produces a duplication of the body axis [145]. Thus, CYP26 enzymes play a very early and important role in regulating access of the embryo to RA.

### 3.5. RA in the Early Embryo

Using direct identification by HPLC, all-*trans* retinaldehyde is detected in embryos as early as the egg cylinder or pre-primitive streak stage, whereas RA is detected between the mid-primitive streak stage and the late allantoic bud stage [146]. Expression of a *β2-RARE-lacZ* (*RARE-lacZ*) transgene that is activated in reporter mice when retinoid interacts with the RAR, has been used extensively as a marker of retinoid-signaling activity *in vivo* [147]. In the post-implantation embryo, definitive staining is noted throughout the length of the primitive streak and the appearance of staining is coincident with formation of the neural plate. The time of initiation of RA synthesis within the embryo proper has also been deduced from studies of retinaldehyde dehydrogenase mRNA (*Raldh2*). It is first detected at the mid-primitive streak stage adjacent to the node and primitive streak [148,149]. The early activity of *Raldh2* in the embryo, along with expression in the visceral endoderm (extraembryonic) is required for vascular development in both the embryo proper, as well as the yolk sac [150,151,152]. Prior to this time RA in the embryo is believed to be of maternal origin [145]. HPLC studies of embryonic tissues at later times confirm that all-*trans* retinol and RA are the primary retinoids detected, with retinol clearly the most abundant [153,154,155].

The importance of protecting the embryo from RA at times when it is not needed is highlighted by recent work on embryos null for all three CYP26 family members. RA is available from the maternal circulation, and *Raldh2* is expressed in the endometrial stroma and decidua [156]. *Raldh2* is highly expressed in the endometrial stroma by E2.5, remains high after implantation, and expression is reduced as stromal cells undergo decidualization. Uehara *et al.* report that the developing embryo is protected from maternal sources of RA by *Cyp26* family members expressed in extraembryonic cell types including extraembryonic endoderm and ectoderm, visceral endoderm and ectoplacental cone that surround the murine embryo proper at E5.5 and E6.25 [145]. When all three CYP26 family members are deleted, embryos show a duplication of the body axis resulting from expanded *Nodal* expression driven by RA throughout the epiblast. *LacZ* staining that results from RA-driven expression of the *RARE-lacZ* reporter is not normally detected in wild-type embryos at E6.25, but is abundantly expressed in CYP26A1/B1/C1 triple knockout embryos. Thus, the CYP26 catabolic enzymes play a key role in regulating the exposure of the early embryo to maternal RA.

### 3.6. Early Nervous System Development

Vitamin A is required for many aspects of nervous system development including patterning and neural differentiation [157,158]. One of the best-studied functions of RA is in the developing hindbrain where it contributes to the anteroposterior patterning of the neural plate [159]. When induced, the neural plate is initially anterior or forebrain-like. Induction of more posterior domains occurs by the actions of RA, Wnts and FGFs, with RA specifying the posterior hindbrain and cervical spinal cord [160]. Patterning in the vertebrate hindbrain involves segmentation, a strategy that is used to direct the diverse range of nerves and craniofacial structures essential to hindbrain function [161]. Hindbrain segments called rhombomeres (r), are transient structures that express a distinct set of cellular and molecular properties, including an ordered pattern of *Hox* gene expression. Using the VAD quail model, the Maden and Zile laboratories first observed that the posterior region of the hindbrain (rhombomeres 4-7) does not develop in VAD embryos [162]. The effect of depleting vitamin A by a number of means in a variety of organisms was then studied in great detail. Loss of function mutation of RALDH2 in both mice and zebrafish results in anteriorization of the hindbrain [163,164,165]. VAD rat embryos generated by severely restricting the amount of RA fed to deficient mothers at the beginning of pregnancy results in a loss of posterior rhombomere segmentation, with a significant shortening of the hindbrain [65]. In addition, ectopic otic-like vesicles appear posterior to the orthotopic vesicle. By varying the amount of RA added back to the maternal diet, this study provided evidence that an increasing amount of retinoid is needed for the correct specification of more posterior rhombomeres. 

RARα/γ compound mutants show a hindbrain phenotype similar to that seen in vitamin A deficiency [166], whereas the RARα/β compound null mutants present a less severe phenotype [167], indicating that RARα and RARγ play an important role at early stages of hindbrain patterning. Elimination of all RARs in zebrafish severely disrupts hindbrain patterning [168]. Using cultured chick embryos treated with RAR antagonist at various stages of development, distinct developmental time windows were identified when RA specifies specific rhombomeres in a rostrocaudal sequence [169]. 

**Figure 4:**
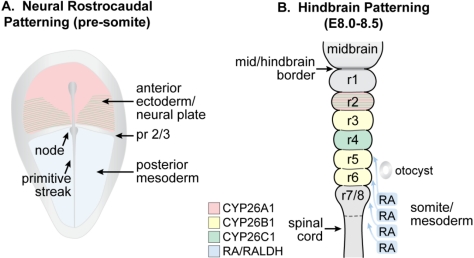
Schematic showing the location of RA and *Cyp26* expression in a presomitic mouse embryo undergoing (**A**) patterning of the neural plate and (**B**) later during the course of hindbrain patterning. (**A**) RA generated by RALDH2 in the posterior mesoderm forms an early anterior boundary of activity in the neural plate at presumptive rhombomere (pr) 2/pr3, whereas *Cyp26A1* and *Cyp26C1* are expressed rostral to the pr2 border; (**B**) By E8.0 to E8.5, RA is being expressed by the somites and anterior presomitic mesoderm, and acts on the overlying hindbrain and spinal cord. The activity of the CYP26 enzymes regulate access of the neuroepithelium to RA (Adapted from [139]).

The RA needed for antero-posterior patterning is produced in the anterior paraxial mesoderm by RALDH2 and diffuses into the adjacent central nervous system (Figure 4A). After this, *Raldh2* mRNA is found in somitic as well as presomitic mesoderm, but not in the node [170]. The generation and diffusion of RA has been proposed to form a gradient (higher caudal/lower rostral) that patterns the hindbrain. However, the ability of exogenous RA to rescue development implies that mechanisms other than simple diffusion of RA from a localized posterior source must be involved in generating differential responsiveness along the hindbrain anterior to posterior axis. 

The importance of CYP26 family members in restricting access of the developing hindbrain to RA was first exemplified by genetic ablation of CYP26A1, resulting in hindbrain posteriorization. The rhombomere-specific expression pattern of the *Cyp26* mRNAs led to the proposal that boundaries of RA activity are created by their expression [23,141,171] (Figure 4B). For example, *Cyp26A1* is initially expressed in the anterior epiblast and neural plate [18] and forms a boundary at presumptive r2/r3 [141]. This is followed by the later expression of *Cyp26A1* in r2 and *Cyp26C1* in r2 and r4 [18,21,139,144]. Accordingly, CYP26A1 mutants show hindbrain abnormalities just rostral to the region affected by retinoid deficiency, including posterior transformation of r2/3 to a r4-like character [139,140], however, individual CYP26B1 and CYP26C1 mutants develop normally [143,144]. A series of detailed studies using mouse embryos carrying the *RARE-lacZ* transgene led to a further posit that the initial gradient of RA entering the posterior hindbrain is converted into RA boundaries that shift over time [172]. It was proposed that CYP26C1 could play a role in restricting RA activity to the rhombomere 4/5 border, although CYP26C1 null mutants reportedly do not show abnormalities at this boundary as assessed by *HoxB1* and *RARE-lacZ* staining [144]. However, CYP26A1/C1 double knockouts do exhibit even more severe hindbrain abnormalities than CYP26A1 mutants alone, including a grossly enlarged posterior hindbrain at the expense of anterior structures [144]. Interestingly, in *Cyp26* morpholino studies in zebrafish, restriction of the RA-responsive gene, *Vhnff1*, is determined by the posterior limit of *Cyp26C1* at the r4/r5 boundary, a function that can be compensated for by CYP26B1 [173]. Thus, the combined action of these enzymes along with the dynamic changes in where they are expressed during development must prevent RA from reaching specific anterior structures. Although not identical, recent models explaining how RA patterns the developing hindbrain all include an important role for CYP26 family members in regulating the formation of waves or pulses of RA in this dynamic process [23,144,172,173]. 

When hindbrain patterning is underway, exposure of the anterior brain to RA is prevented by the action of CYP26A1 and CYP26C1, and loss of CYP26A1 renders embryos more sensitive to the teratogenic effects of excess exogenous RA on head truncation [174]. By regulating levels of RA in the anterior brain, the CYPs may enable a ligand independent function of the RARs. Based on studies in *Xenopus* embryos, unliganded RARs in the head region at this early time are proposed to function as transcriptional repressors, that if activated inappropriately by RA, lead to activation of target genes that should remain off [175]. Thus, teratogenesis may result from over activation of genes that are normally upregulated by RA and/or a loss of gene repression by unliganded RAR.

A role for RA in forebrain patterning was initially proposed based on studies in chick embryos exposed to RAR and RXR antagonists [176] and from the VAD quail model in which the size of the telencephalic vessel is reduced and the diencephalon is abnormally patterned [177]. Additional studies in VAD quail indicated that the vitamin was needed to correctly position anterior and dorsal boundaries in the forebrain via modulation of FGF8 and Wnt signaling [178]. RA was also reported in chick to contribute to regionalization of the telencephalon along the dorsoventral axis, and RALDH3 produced by the head ectoderm was proposed as the retinoid source [179]. However, RALDH2/3 compound null mutant mice reportedly do not show defects in the early molecular determinants of forebrain patterning [178,180]. Instead, Molotkova and colleagues propose that RA functions at later times in forebrain development, and provide evidence that RA generated by RALDH3 in the lateral ganglionic eminence is required for normal expression of the dopamine D2 receptor in the nucleus accumbens and medial striatum [180].

Additional roles for RA in later development of the central nervous system (CNS) are beginning to emerge [181]. McCaffery’s group found that RA is produced by RALDH2 in the meninges overlying the hindbrain at mouse embryonic day 13, and that RA activity as assessed by *RARE-lacZ* is present in precerebellar neurons migrating around the hindbrain circumference to form the inferior olive and pontine nuclei [182]. This group went on to show that RA is required for the generation of posterior neurons in the inferior olive, as the posterior inferior olive is significantly smaller in late VAD rat embryos compared to vitamin A-sufficient controls [183]. In the meninges covering the cortex, levels of *Raldh2* increase in the mouse embryo from E13 to E14 onward, to a maximal level in the newborn brain [184]. Recently, RA from the meninges was reported to function in cortical development by regulating the switch of radial glia cells in the ventricular zone from symmetric to asymmetric division, resulting in the formation of neurons or intermediate progenitor cells [185]. It will be important to determine whether RA regulates stem cell division/differentiation in any other areas of the CNS.

### 3.7. Spinal Cord and Other Neuronal Development

RA plays a role in the development of caudal structures, including the neural tube that forms the spinal cord and in somite development. These tissues arise from the node-streak border, a region that comprises the caudal end of the node and the rostral end of the primitive streak [186]. Although primary neurulation occurs in both RALDH2 mutant mice and VAD chick and rat embryos [65,123,187], the RALDH2 mutant is reported to have a thinner neuroepithelium [149] and VAD quail embryos have fewer spinal cord neurons and smaller somites [162,188]. *Raldh2* is first expressed during gastrulation in the mesenchyme adjacent to the node and the primitive streak [148]. In the mouse and chick embryonic axis, RA from the anterior presomitic mesoderm and somites promotes neural differentiation by inhibiting the expression of *Fgf8*[189]. In RALDH2 mutants, primitive streak and mesodermal markers are expanded at the expense of those representative of the prospective neuroepithelium [149]. 

RA from the adjacent somites is needed to generate certain interneurons and ventral spinal cord motor neurons [190,191]. RA generated from within the spinal cord is required for the differentiation of a subset of lateral motor column (LMC) neurons that extend axons into the limb [192,193]. Interactions between HOXD10, which defines rostral boundaries of the lumbosacral LMC, and HOXD11, which opposes the effects of HOXD10, influence the distribution of motor neuron subtypes along the rostrocaudal axis. HOXD11 may also serve to restrict RALDH expression to regions where it is needed to support LMC neuron survival and maturation [194]. 

There is evidence that RA promotes the survival of sympathetic neurons by inducing responsiveness to neurotrophins [195,196,197]. RA is also linked to the promotion of neurite outgrowth [157,162]. Recently, a number of genes including neuron navigator 2 (*Nav2*) and *Nedd9* were discovered in a screen for RA-responsive genes in a neuroblastoma cell line that elaborates neurites in response to RA [198,199,200]. *Nedd9* is a direct downstream target of RA, whereas a functional RARE has not yet been identified in the *Nav2* gene [33]. When *NAV2* is knocked down, the neuroblastoma cell is no longer able to extend neurites in response to RA [201]. The human *Nav2* gene can rescue defects in axonal elongation in the *C. elegans* mutant, UNC-53 (the homolog of *Nav2* in the worm), and a mouse mutant hypomorphic for *NAV2* also shows defects in cranial nerve development and general neurite density [202]. *NAV2* may function as a link between actin remodeling and microtubule dynamics during neurite outgrowth.

### 3.8. Eye Development

Recognition of the importance of vitamin A in eye development first came from the experiments of Hale in which piglets from deficient mothers were born blind [86,87]. The morphologic consequences of vitamin A deficiency in rat embryos were examined in great detail by Warkany and Scharffenberger [61,88] and Wilson *et al.* [95]. They noted dysmorphogenesis of the anterior eye segment, retina and optic disc, although the penetrance of the defects was extremely variable. Using a model in which normal development is supported in VAD rat embryos up to embryonic day 10.5 (12-15 somite stage) with RA and deficiency is induced thereafter, See *et al.* was able to produce eye defects in 100% of embryos which included; coloboma, absence of the anterior chamber of the eye, rudimentary iris or loss, fusion of the lens and cornea, thickening of the eyelid tissues, reduced size of the conjunctival sac, folding of the retina, absence of the vitreous body and the presence of a fibrous retrolenticular membrane [67,104]. The addition of retinol at E10.5 provided a full rescue of all defects, showing that all the defects were attributable to retinoid deficiency imposed after this time. The addition of a high level of RA after E10.5 dramatically improved eye development, supporting that vitamin A acid is the active moiety. 

The RA synthesizing enzymes, RALDHs, show a very dynamic pattern of expression in the developing eye. In the mouse, *Raldh2* (also called V2 activity) is transiently expressed in the optic vesicle neuroepithelium at E8.5 [132,148,203], after which *Raldh3* (also called V1 activity) is expressed in the ventral neural retina and pigmented neuroepithelium [134,204,205,206] and *Raldh1* (also called ADH-2) is expressed in the dorsal retina from E9.5 onward [134,207]. RARα is expressed in all layers of the developing murine neural retina, whereas RARβ is expressed in the inner nuclear layer from embryonic day 14 to postnatal day 7 [208,209]. Genetic ablation of two or more retinoid receptors in mice results in eye defects that are largely similar to those seen in VAD rat embryos [97,209,210,211]. A number of eye defects in RALDH3 and RALDH1/3 compound mutants [134,135,178] are also similar to those observed in late VAD rat embryos.

Using the late VAD rat embryo model, it is possible to identify the times when vitamin A must be present to support eye development by adding back retinol at successively later times ranging from E11.5 to E15.5, and evaluating eye development at E18.5 [104]. A full rescue of all structures is achieved if retinol is added by E11.5, whereas addition on or after E14.5 is ineffective. Retinol added as late as E13.5 completely prevents retinal folding, formation of a fibrous retrolenticular membrane, and loss of the vitreous body but is ineffective in preventing anterior eye segment defects, whereas addition by E12.5 improves or rescues the majority of defects in anterior eye segment development (absence of anterior chamber, rudimentary iris, corneal-lenticular stalk fusion, and small conjunctival sac) and prevents coloboma of the retina and optic disc in the majority of fetuses. This study also revealed that the cells within the retina of late VAD embryos lose their characteristic shape and orientation along the apical-to-basal axis of the retina, with the appearance of gaps or holes in the neural retina that worsen as retinol is added at successively later times. Interestingly, the increase in cell adhesion proteins, *N*-cadherin and β-catenin, that normally occurs with development is not seen in VAD retinas. Additionally, a reduction in cyclin D1 labeling is observed in the retina of late VAD fetuses, suggesting that cell proliferation is also disrupted. These effects may contribute to retinal collapse seen in VAD rat embryos receiving no supplemental retinol, or retinol on or after E14.5. Using this temporal model, See *et al.* also clearly showed that a lack of optic fissure closure does not lead to retinal folding/collapse, as a well formed retina is produced by adding retinol at a time that is too late to prevent coloboma [104]. Retinal thinning and disruption of cellular organization and adhesion is a more likely reason for collapse of the retina seen at late stages of severe deficiency. 

**Figure 5:**
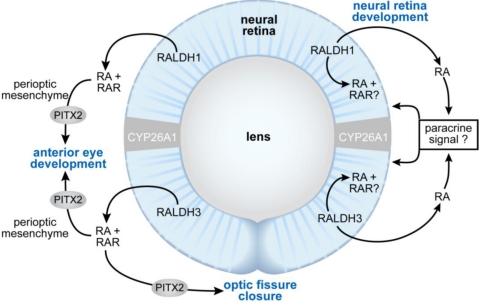
Schematic showing the proposed sites of RA function during eye morphogenesis (left) and differentiation (right). At early stages of eye development, RA generated by RALDH1 and RALDH3 acts as a paracrine signal binding to RARs located in the perioptic mesenchyme to support anterior eye segment development and closure of the optic fissure. *Pitx2* is a RA/RAR-regulated transcription factor that is required both for anterior eye segment morphogenesis, as well as closure of the optic fissure. At later stages of development, RA promotes differentiation of the neural retina. The mechanism is unclear, but could involve either a paracrine effect of RA outside of the neural retina, or a direct effect on the cells within the retina itself.

RARα has been reported to be the sole receptor transducing the RA signal in the retina beyond E10.5 based on the inability to detect *RARE-lacZ* expression in the RARα null mutant mouse retina [212]. In the absence of this receptor, however, the mouse retina appears to develop normally leading to the conclusion that RARα is unnecessary for the developing mouse retina [213]. If *RARE-lacZ* expression is an accurate readout of all RAR-mediated signaling in the neural retina of RARα null mutants, then the neural retina would appear not to be a direct target of RA and its receptors. If so, then it is possible that adverse effects of vitamin A deficiency on neural retina development could occur by a loss of paracrine action of RA in a secondary tissue, as is proposed for retinoid support of anterior eye segment development (Figure 5). Thus, it is unknown whether RA generated in the retina acts locally to regulate retina development.

The molecular basis for the vitamin A deficiency-induced defects in anterior eye segment development and in the appearance of coloboma has been the subject of several recent studies. It does not involve abnormalities in dorsoventral patterning genes, but rather, disruption of paracrine signaling from the retina to the perioptic mesenchyme. Compound deletion of both RARβ and RARγ in the neural crest cell-derived periocular mesenchyme using *Wnt1-cre* to direct excision results in eye defects including abnormalities of the cornea and anterior chamber [134,212]. Similarly, RALDH1/3 compound null mutants show malformations of the anterior segment of the eye, including agenesis of the corneal and iris stroma, agenesis of the anterior chamber and less frequently, coloboma of the retina [134,135]. Thus, RA from retina is believed to signal in paracrine fashion to the perioptic mesenchyme to support the development of anterior eye structures. Defects result from the loss of control of programmed cell death in this region, and by loss of RA-mediated *Pitx2*, *Foxc1*, *Eya2* and *Dkk2* expression, and subsequent loss of repression of Wnt/β-catenin signaling [134,135,212,214]. *Pitx2* encodes a homeodomain transcription factor that is essential for anterior eye segment development, and is also required for optic fissure closure [215]. *Pitx2* is down regulated in the periocular mesenchyme of late VAD embryos that not only show defects in anterior eye segment development, but also retinal/optic disc coloboma at 100% penetrance [67,104]. Very recently it was shown that the *Pitx2* gene contains a RARE located 4.3 kb upstream of the promoter, and thus is a direct target of RA and its receptors [214]. Duester and colleagues propose that RA signaling activates *Pitx2* in the perioptic mesenchyme, which in turn, induces *Dkk2* and suppresses local Wnt signaling in the regulation of normal anterior segment development. Zacharias *et al.* has extended this model to include a role for canonical Wnt signaling in the maintenance of *Pitx2* expression after its initiation by RA, and prior to the time that Wnt signaling is suppressed [216].

### 3.9. Somites and Skeleton

RA plays a role in the initiation of differentiation of the anterior region of the presomitic mesoderm from which the new somites originate [217]. VAD quail embryos have smaller somites than their vitamin A-sufficient counterparts, and also have an expanded FGF8 domain in the presomitic mesoderm [188]. In RALDH2 null mutants, the expression of *Fgf8* mRNA in the primitive ectoderm (epiblast) is shifted anteriorly such that it enters the node ectoderm and neuroectoderm, and somite development is abnormal [170]. FGF signaling is important in the process of somitogenesis, and the opposition of FGF and RA signaling is also important in regulating somite size. A reduction in FGF in the presomitic mesoderm below a threshold value is needed to position the future somite boundary [218,219]. Higher levels of FGF8 and Wnt3a signaling maintain cells in an undifferentiated state, whereas exposure to RA along with lower FGF8 and Wnt3a initiates differentiation, and these factors are believed to control the developmental switch at this boundary [217]. Recent work in chick indicates that RA may also be involved in the termination of the process of segmentation [220].

RA signaling is involved both in the specification of the axial identity of future somites as well as in later stages of skeletal development. Somites, although similar by appearance, develop into distinct structures dependent upon their axial position. Determination of initial axial identity is believed to occur in mesodermal cells prior to somite formation [221]. VAD rat embryos show anterior vertebral transformations throughout the axial skeleton that can be rescued only if vitamin A is provided on or before embryonic day 8.75, a time that precedes appearance of the first somite [66]. Genetic deletion of several RARs produces defects in axial development with anterior cervical transformations similar to VAD embryos [53,97,209]. However, in these mutants, either posteriorization at the cervical/thoracic junction is observed, or no vertebral changes caudal to this region are reported [97,209]. Alteration of *Hox* gene expression appears to be major way in which retinoids affect positional information along the anteroposterior body axis [222,223,224,225].

Vitamin A is also required for skeletal development beyond its role in presomitic embryos. Rat embryos given sufficient RA up to embryonic day 10.5 (approximately the 12-15 somite stage), but made deficient thereafter exhibit hypoplastic cranial bones, defects of the thyroid, cricoid and tracheal cartilages as well as agenesis of the neural arch of cervical vertebrae 1 (C1) and ectopic bone in the dorsal regions of C1 [67]; defects in this region bear many similarities to those observed in RARα/γ compound null mutants [97,98]. Late VAD rat embryos also exhibit gross malformation of the sternal and pelvic regions [67]. Sternal malformations have been reported in RBP null mutant embryos from RBP null mothers fed inadequate vitamin A [110]. Surprisingly, late VAD rat embryos have anterior vertebral transformations in the cervical axial skeleton up to and including the thoracic juncture, along with posteriorization events at the thoracic and sacral levels of the skeleton [67]. As discussed above, a number of RAR mutants show both cervical anteriorizations and posteriorization at the cervical thoracic junction [97,209]. It is also interesting that administration of excess RA to the mouse at E7 (presomitic; equivalent to E8.5-9 rat) produces posteriorizing transformations throughout the skeleton; however, excess RA at E8.5 (equivalent to E10-10.5 rat), produces anterior transformations starting at vertebrae 15 (thoracic vertebra 8, T8) whereas excess RA at even later times, yields both rostral posteriorizing and caudal anteriorizations [223,224]. Thus, vertebral identities, initially specified at the late primitive streak phase, can also be respecified at later times in mouse development when the vertebrae precursors (the somites), differentiate, and the sclerotome cells begin to form vertebrae. In summary, vitamin A plays a normal role in the maintenance of vertebral identity as well as in the development of skeletal elements.

Much of the craniofacial skeleton originates from neural crest cells [226]. Frontonasal agenesis has been reported in compound RAR null mutant mice [97] and RBP null mutant mice on a vitamin A-deficiency diet [110]. Dupe and Pellerin recently showed that selective ablation of RARα and RARγ subtypes using the *Wnt1-Cre* promoter leads to agenesis of the frontonasal skeletal elements, but does not appear to adversely affect the early survival and migration of neural crest cells [227]. Deletion of all three RAR subtypes using the selective promoter produces a similar phenotype, indicating that RARα and RARγ act cell-autonomously in neural crest cells to direct morphogenesis of these skeletal elements. 

### 3.10. Heart Development

Cardiac and aortic arch defects were observed as a part of the early vitamin A deficiency syndrome in rat embryos [93,94] and in RAR compound null mutant mouse embryos [98,102]. When severe VAD is imposed in quail, the initiation of heart morphogenesis is disrupted [88,228,229]. In VAD quail embryos, vascular networks are absent and the heart appears ballooned and non-compartmentalized, and is randomly-positioned without an inflow tract at the posterior site [228]. Inability of the heart tube to undergo looping is also observed in RALDH2 mouse mutants [230]. The sinuatrial (venous) valve, a transient structure that flanks the orifice between the sinus venosus and right atrium, is not formed properly in rat embryos deprived of RA for one day (E9.5-10.5), and results in improper channeling of venous blood and anterior cardinal vein distension [64]. If RA deficiency is imposed after E10.5 in the rat embryo, a time when the cardiac primordium has completed looping and the primitive vasculature is established, early cardiac and aortic arch defects are not observed [67]. In summary, roles for vitamin A in mammalian heart development include: heart tube patterning and looping, chamber and outflow tract septation, ventricular trabeculation, cardiomyocyte differentiation and coronary vessel development [231,232,233]. A more in depth discussion of the molecular pathways involved in heart morphogenesis can be found in several recent reviews [229,232,234].

### 3.11. Kidney and Urinary Tract Development

The requirement for RA in the developing kidney and urogenital tract has been illustrated in several animal models. In rodents, maternal vitamin A deficiency results in embryonic renal hypoplasia, the severity of which depends on the extent of vitamin A deprivation [67,91,110,235]. Ectopic kidneys, renal fusion and failure of the renal pelvis and calyx to undergo dilatation, in addition to ectopic ureteric openings and other genitourinary tract defects have been reported in VAD rat fetuses [67,91]. RALDH2 null mutant mice lack nephric ducts [123] and in RARα/β2 double receptor mutants, nephron progenitors, stromal cells and ureteric bud tips are all greatly reduced or completely absent at birth [98,236]. These compound RAR mutants also have incorrectly positioned distal ureters, hydronephrosis and megaureter.

Signaling between the ureteric bud epithelium that forms the collecting duct system, the metanephric mesenchyme that differentiates into nephron, and the stromal mesenchyme that differentiates into the renal interstitium is important during early kidney development. In the embryonic kidney, *Raldh2* is localized in stromal mesenchyme of the outer cortex and *Raldh3* is expressed in the ureteric bud [130,237,238]. RA and its receptors are needed to maintain *Ret* expression [237], a gene critically required for formation of the ureteric bud and its branching in the kidney [239,240,241]. The forced expression of *Ret* in ureteric bud cells in RARα/β2 double receptor mutants rescues renal development, restoring ureteric bud growth and stromal cell patterning [237]. Recent work shows that RA generated by RALDH2 in stromal mesenchyme acts in paracrine fashion to activate RA-receptor signaling and *Ret* expression in ureteric bud cells [242]. 

In kidney, the number of embryonic branching events determines the final number of nephrons an individual will have for life, and it has been proposed that suboptimal nephron number at birth increases susceptibility to acquired renal disease and essential hypertension later in life [243,244,245,246]. Studies in rodents suggest that even mild vitamin A deficiency (a 50% decrease in circulating vitamin A concentrations) can lead to impaired branching and a 20% reduction in the number of nephrons [235]. Quite remarkably, a single injection of RA to a control group of pregnant rats at midgestation (E11) led to supernumerary nephron endowment in the kidneys of their offspring. RA given intraperitoneally at E11.5 is also able to increase nephron endowment in offspring exposed to maternal protein restriction without affecting body weight or kidney size, such that the number of nephrons per volume of kidney tissue is increased in these rat pups above that seen in the kidneys of control offspring [247]. Hence mild vitamin A deficiency in pregnancy may correlate to sub-clinical deficiencies in nephron number and slight nephron deficits that are not recognized at birth, but could possibly contribute in the long-term to renal failure and hypertension. 

### 3.12. Diaphragm

The diaphragm functions as the primary muscle of respiration and forms a physical barrier between the thoracic and abdominal cavities. Congenital diaphragmatic hernia (CDH) occurs in approximately one in 3000 births, and is associated with high neonatal mortality [248]. Vitamin A is essential for normal diaphragm development, and it has been hypothesized that disruption of retinoid signaling may contribute to the etiology of the human disorder. A recent report shows that there is a relationship between low cord retinol and RBP levels and CDH in newborn infants [249]. 

A herniated diaphragm was observed as a part of the early vitamin A deficiency syndrome [89], and appears at a 100% penetrance in RA-supported VAD embryos given insufficient RA after E10.5 of development, whereas a normal diaphragm results if the mother is supplemented with retinol after this time [67]. Diaphragmatic hernia is also observed at low penetrance in RARα/β2 compound null mutant mice [98]. Congenital diaphragmatic hernia in humans is linked to mutations in *Stra6*, which encodes for a membrane RBP receptor [26,250], as well as to CDH-affected chromosome loci encoding for several other retinoid-related genes [251]. 

The pleuroperitoneal fold (PPF) is a transient structure formed at the union of the pleuro-pericardial folds and the septum transversum and represents the component of the primordial diaphragm through which muscle precursor cells and pioneer axons of the phrenic nerve migrate to form the mature diaphragm. The PPF is fully formed by E13.5 in vitamin A-sufficient rat embryos, but it is abnormal in late VAD embryos [252]. Similar defects in PPF development are seen in rat embryos treated with nitrofen and several other CDH-inducing teratogens that interfere with the synthesis of RA and RA signaling *in vivo* [253,254]. *Raldh2* mRNA is expressed in the PPF, and RARs α, γ and RXRα are most strongly expressed in the nonmuscular mesenchymal cells of the PPF. Thus, vitamin A signaling in the developing PPF appears to play a key role in the developing diaphragm.

### 3.13. Lung and Upper Respiratory Tract and Airways

Respiratory defects including left lung agenesis, bilateral lung hypoplasia, and agenesis of the esophagotracheal septum were described in early VAD syndrome embryos but were characterized as rare anomalies [90,95]. Lung hypoplasia is observed with 100% penetrance in rat embryos in which RA deficiency is imposed after E10.5, and the severity is increased as RA in the maternal diet is reduced [67]. *Rars* are expressed throughout lung development and RAR compound null mutant mice (RARα/β) show left lung agenesis and hypoplasia [209]. Additionally, a wide array of RA synthesizing, metabolizing and binding proteins are found in developing lung [255,256,257]. Mice null for RALDH2 or RDH10, also have lung agenesis or hypoplastic phenotypes [122,258,259]. The lung is second only to the liver as the major retinoid storage organ [260].

The lung arises from foregut endoderm during early development of the embryo. RA from the splanchnic mesoderm surrounding the foregut endoderm has been found to be essential for primordial lung bud formation at E9.5 in the mouse [256,258,259,261]. It was recently shown that in the foregut mesoderm, RA controls the *Fgf10* expression required for bud formation by balancing the activation of canonical Wnt signaling through direct transcriptional repression of its antagonist *Dkk1*, and repression of Tgfβ signaling [262]. This conclusion is reinforced by work showing that simultaneous activation of Wnt and repression of Tgfβ in RA-deficient foregut rescues lung bud formation. 

**Figure 6:**
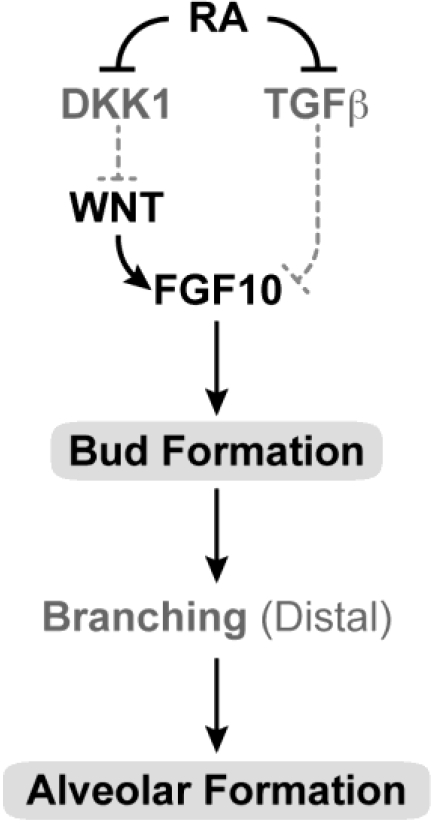
Schematic showing the proposed sites of RA action in lung development. In the developing embryo, RA is needed for primary bud formation (grey oval), but signaling is down-regulated during the time that lung differentiation occurs. A later role for RA in alveolar formation (grey oval) is also proposed. During induction of the lung buds, RA regulates mesodermal *Fgf10* levels by negatively regulating Tgfβ and enabling induction of the Wnt pathway by repression of the Dickkopf homolog 1 (*Dkk1*) known to antagonize Wnt ligand-receptor binding. RA may also influence the response of the foregut endoderm (origin of lung progenitors) to *Fgf10*. (Adapted from [262]).

While RA signaling is required for initial budding, by E10.5-E11.5, as secondary buds form, levels are down regulated by the appearance of the RA-degrading enzyme CYP26A1 to enable more distal branching and distal airway formation to proceed to completion [256,263]. A role for RA in alveoli formation is supported by the finding that RARs are required for correct lung alveoli septation, and reports that exogenous RA can stimulate alveoli formation in immature rat and mouse lung [264,265,266,267,268,269]. Figure 6 summarizes the proposed functions of RA in lung development.

A recent report in the New England Journal of Medicine showed that, in a region with endemic vitamin A (retinol) deficiency, children whose mothers had received vitamin A supplementation before, during, and for 6 months after pregnancy had better lung function when they were tested at 9 to 11 years of age than children whose mothers had received beta carotene supplementation or placebo. Additionally, they found that the period during which supplementation with vitamin A was most important was from gestation through a postnatal age of 6 months [270].

RA acting through the RAR is also necessary for the morphogenesis of other components of the respiratory tract including partitioning of the primitive foregut into oesophagus and trachea, and for the opening between the nasal and oropharyngeal cavities [67,131,209,256,258,261].

### 3.14. Pancreas

Although not observed either as a part of the vitamin A deficiency syndrome or in compound RAR null mutant mice, a requirement for RA in pancreatic development has been proposed based on studies in RALDH null mutant mice [271,272] and in several other organisms in which retinoid signaling is deficient or inhibited, including *Xenopus*, zebrafish, chick, and quail [273,274,275,276]. RDH10 knockout mice do not have a pancreas [122]. RA produced by RALDH2 is required for early development of the dorsal pancreas in the mouse [271,272]. Specification of dorsal pancreatic tissue can be rescued in RALDH2-deficient embryos by low-dose maternal administration of RA. 

The effects of RA deficiency on pancreatic lineages appear to be due to the loss of pancreatic field specification within the endoderm [274]. Specification of the pancreas occurs between E8.0-8.5 of mouse development, and is followed by the development of the dorsal and ventral pancreatic buds at E9.0-9.5 [271]. RALDH2 null mutant embryos do not develop a dorsal pancreatic bud. It has been suggested that the differential expression of retinoic acid receptors (RARs) in gastrula stage endoderm is at least partially responsible for the distinct responsiveness of dorsal *versus* ventral pancreas [277]. Both defects in RA signaling and RA treatment have been shown to affect the expression of PDX-1 [271,272,275,278] an essential regulator of early pancreas development required for the pancreatic buds to grow and differentiate [279,280,281]. RALDH2 mutant embryos lack PDX1 expression in dorsal but not ventral endoderm [271,272]. Conversely, exogenous RA has been shown to expand the pancreatic field [273,274,275], and CYP26A1 has recently been shown to play a critical role in setting the anterior limit of the pancreas field endodermal *Cyp26* expression [282]. Additional information regarding the role of RA in pancreas development can be found in several recent reviews [283,284].

### 3.15. Limb Development and Interdigital Cell Death

Functions for RA in limb development have been forwarded and debated for many years. Inhibitors of RA synthesis and vitamin A deficiency inhibit limb outgrowth in the quail [285,286]. In the absence of RALDH2, murine forelimb buds do not develop and embryonic growth ceases prior to the stage when hindlimb buds are initiated [287,288]. Zebrafish RALDH2 mutants lack pectoral fins and fin bud induction does not occur [164,165,276,289]. Limb defects in mice also result from treatment with excess exogenous RA, or inappropriate exposure to endogenous levels of RA due to the absence of CYP26B1. These studies show that controlled exclusion of RA from the limb bud is essential for proper limb morphology [142]. 

RA has been proposed to play a role in patterning the developing limb by regulating the pattern of expression of genes such as *Hand2* (activates SHH), *Shh* (patterns the anterior-to-posterior axis), and *Meis1/2* (transcription factors that mark the proximal limb bud mesenchyme) [287,290,291,292]. However, a new report from Zhao and colleagues using a RALDH2/3 double mouse knockout suggests that RA acts as a permissive signal at an earlier stage to allow limb bud initiation rather than acting in an instructive manner, and that the role of RA is to antagonize early axial FGF signals which otherwise inhibit the limb field [80,141,293]. According to this new model, RA signaling within the forelimb bud proper is not required for normal patterning to occur. A similar idea was proposed by Gilbert *et al.*, who found that axial retinoic acid signals played a permissive role in the induction of zebrafish pectoral fins [289]. RA appears to be dispensable for hindlimb budding and patterning [80,287]. It is notable that trex embryos carrying a mutation in *Rdh10*, have small, abnormal forelimbs but have hindlimbs that are relatively unaffected [122].

At later stages of development, RA is also essential for interdigital cell death, the mechanism by which digit separation occurs. Webbed digits have been described in compound RAR mutant mice due to a loss of apoptosis [209,294]. Around E12.5 (mouse) RA signaling becomes confined to the interdigital zones by a combination of interdigital *Raldh2* expression and *Cyp26B1* expression in the developing digits [295]. The mechanism by which RA activates cell death is currently a subject of active investigation [294,295,296,297]. 

## 4. Conclusions

In summary, the vitamin A metabolite, RA, is essential for reproduction in both the male and female, as well as for many events in the developing embryo. Nutritional as well as genetic approaches are being used to identify the cell types and pathways that are dependent upon RA signaling in support of these processes. Paracrine signaling appears to play a prominent role in RA action. Regulation of RA synthesis as well as its catabolism is important in determining when and where RA signaling will be activated. Future studies are needed to develop a more detailed understanding of when in development, and in what specific cell types RA and its receptors are acting. Elucidation of the pathways that are involved in support of vitamin A functions in stem/germ cell division/differentiation, patterning and tissue/organ development remain major tasks for future work. 
